# Hypnosis for treatment of insomnia in school-age children: a retrospective chart review

**DOI:** 10.1186/1471-2431-6-23

**Published:** 2006-08-16

**Authors:** Ran D Anbar, Molly P Slothower

**Affiliations:** 1 Department of Pediatrics, University Hospital, State University of New York Upstate Medical University, Syracuse, NY, USA

## Abstract

**Background:**

The purposes of this study are to document psychosocial stressors and medical conditions associated with development of insomnia in school-age children and to report use of hypnosis for this condition.

**Methods:**

A retrospective chart review was performed for 84 children and adolescents with insomnia, excluding those with central or obstructive sleep apnea. All patients were offered and accepted instruction in self-hypnosis for treatment of insomnia, and for other symptoms if it was felt that these were amenable to therapy with hypnosis. Seventy-five patients returned for follow-up after the first hypnosis session. Their mean age was 12 years (range, 7–17). When insomnia did not resolve after the first instruction session, patients were offered the opportunity to use hypnosis to gain insight into the cause.

**Results:**

Younger children were more likely to report that the insomnia was related to fears. Two or fewer hypnosis sessions were provided to 68% of the patients. Of the 70 patients reporting a delay in sleep onset of more than 30 minutes, 90% reported a reduction in sleep onset time following hypnosis. Of the 21 patients reporting nighttime awakenings more than once a week, 52% reported resolution of the awakenings and 38% reported improvement. Somatic complaints amenable to hypnosis were reported by 41%, including chest pain, dyspnea, functional abdominal pain, habit cough, headaches, and vocal cord dysfunction. Among these patients, 87% reported improvement or resolution of the somatic complaints following hypnosis.

**Conclusion:**

Use of hypnosis appears to facilitate efficient therapy for insomnia in school-age children.

## Background

Insomnia is defined as difficulty in falling asleep, prolonged nighttime awakenings, or insufficient amount or quality of sleep, which affects daytime functioning, despite adequate opportunity for sleep [1,2]. Daytime somnolence associated with insomnia in childhood and adolescence may cause impairment in attention, cognition and memory, which can adversely impact academic performance [2]. In children, insomnia typically is not a primary condition [2], but rather associated with psychological or medical conditions, such as anxiety [3], depression [4], pain [5], asthma and cystic fibrosis [6].

Insomnia affects a large proportion of school-age children. In a survey published in 1997, parents of 987 New York State elementary school children, ages 5–12 years, reported that 11% of their children had difficulty falling asleep, 7% had nighttime awakenings, 17% had difficulty awakening in the morning, and 17% were tired in the daytime [7]. In a 1994–1995 survey of 2,339 US adolescents, 12–16 years of age, 23% reported difficulty with falling asleep or nighttime awakenings once a week or more, and 39% reported frequently waking tired [8].

Insomnia in elementary-school children often involves complaints about nighttime fears and anxiety-provoking dreams [3,9,10]. Sleep disturbances during adolescence can occur as a result of differing bedtimes on school nights as opposed to non-school nights, inadequate parental supervision, environmental factors including use of television and the internet, and consumption of alcohol or caffeine [2]. Adolescents also experience an apparent biologically based delay in the timing of sleep onset and awakening, associated with their pubertal status, which can cause a conflict with the social demands for early morning schooling [11-13].

Treatments for insomnia include cognitive behavioral therapies and pharmacologic therapies [1,2]. However, there is a lack of data regarding the efficacy of pharmacologic agents in the management of children with insomnia [14]. The following types of cognitive behavioral therapies are appropriate for school-age children:

(1) Stimulus-control therapy is intended to teach the child to associate the bed with sleep. With this modality, the child goes to bed only when sleepy, uses the bedroom only for sleep, has a regular wake time regardless of the duration of sleep, and avoids daytime napping [15].

(2) Sleep restriction therapy initially reduces the amount of time in the bed to the estimated time spent asleep, and then the time is increased in increments every week until optimal sleep duration is achieved [1,2,15].

(3) Relaxation therapies, including imagery training, meditation, hypnosis, progressive muscle relaxation, and biofeedback [1,2].

(4) Education regarding sleep needs and consequences of poor sleep can help decrease anxiety regarding the inability to fall asleep [1,15].

(5) Sleep-hygiene education emphasizes correction of extrinsic factors that may affect sleep, such as pets, television, room temperature, or exercise [1,15].

Most published reports regarding the use of hypnosis as a cognitive therapy for insomnia have involved adults. Instruction of self-hypnosis for relaxation in two sessions to 18 subjects between 29 and 60 years of age was shown to be more effective in improving insomnia than use of nitrazepam or placebo [16]. Among 45 subjects between 23 and 67 years of age, the use of four 30-minute sessions to instruct hypnosis for relaxation and sleep hygiene education was shown to be more effective with insomnia than stimulus-control or placebo therapy [17]. The insomnia of 3 of 6 subjects between 26 and 61 years of age improved following 6 sessions of hypnosis instruction and reinforcement, including two initial sessions that had a total duration of 210 minutes [18]. Single case reports of improvement of insomnia with the aid of hypnosis in children have been published [10,19,20]. Because children are adept at using hypnosis [20], a non-pharmaceutical intervention without side-effects, we chose to study its effect on insomnia in a larger pediatric population.

The purposes of this study are to document psychosocial stressors and medical conditions associated with development of insomnia in a large series of school-age children and to report use of hypnosis for this condition.

## Methods

A retrospective chart review was undertaken for patients treated for insomnia at the SUNY Upstate Medical University Pediatric Pulmonary Center between 1998 and 2005. Insomnia was defined for this study as trouble falling asleep for more than 30 minutes at least once a week, or nighttime awakening at least once a week. Patients reporting poor quality sleep without associated sleep onset delay or nighttime awakening, or those diagnosed as having central or obstructive sleep apnea were excluded.

All 84 patients who reported insomnia were offered and accepted the opportunity to be instructed in self-hypnosis. Of these, 9 were lost to follow-up after the first hypnosis session and were excluded from the analysis. The diagnoses of the remaining 75 patients (57% male) are shown in Table 1, including 39% of the children who had more than one diagnosis. Forty nine percent of the patients were referred for evaluation of pulmonary symptoms. The remaining patients were referred for treatment with hypnosis of non-pulmonary somatic complaints (23%), anxiety (14%), or insomnia (14%). The average age was 12 years (range, 7–17). The average duration of insomnia prior to hypnosis was 3 years (range, 6 months – 5 years). Pharmacologic therapy had been utilized for the insomnia in 11% of the patients, including diphenhydramine (5 patients), amitriptyline (2 patients), clonidine (2 patients), and melatonin (1 patient).

**Table 1:** Diagnoses of children who reported insomnia n = 75

	n
*No additional diagnosis*	11
	
*Pulmonary*	
	
Asthma	22
Vocal cord dysfunction	6
Dyspnea	3
Cystic fibrosis	2
Habit cough	2
Chest pain	1
	
*Other medical*	
	
Headaches	18
Allergies	9
Gastroesophageal reflux	6
Functional abdominal pain	5
Tics	2
Cerebral palsy	1
Osteogenesis imperfecta	1
Ulcerative colitis	1
	
*Psychological*	
	
Anxiety	21
Attention deficit disorder	9
Asperger's syndrome	1
Obsessive compulsive disorder	1
	
29 children had more than one diagnosis	

Information was collected regarding the nature of the insomnia, as described by the children's self-reports at the beginning of the first session of hypnosis instruction (duration of symptom, frequency of difficulty with falling asleep and/or nighttime awakenings), reasons cited by the children for the insomnia, and frequency of the hypnotic intervention. Outcome of the intervention was assessed based on the children's self-reports within a month of the last hypnosis session, and when available, children's reports at later times. Resolution of insomnia was defined as sleep onset delay of less than 30 minutes and decreased frequency of nighttime awakenings to less than once a week. No systematic objective measurement strategy for insomnia was utilized before or after the hypnosis intervention, such as a sleep diary or parental observation. Assessment of intervention effectiveness was based on the patients' subjective reports.

Patients were evaluated for their respiratory complaints by a thorough review of their history, physical examination, laboratory investigations, pulmonary function testing, and radiological imaging. Medical therapy was prescribed for pulmonary conditions, when indicated.

Patients were offered hypnosis for insomnia if they reported this symptom. Also, they were offered hypnosis for respiratory or other symptoms if it was felt that the complaints were amenable to therapy with hypnosis [21-24]. Those who expressed interest were instructed in self-hypnosis techniques by the pediatric pulmonologist. Self-hypnosis instruction typically was provided in one or two 15 to 60 minute sessions.

The first session included: (1) A description of hypnosis; (2) demonstration of two or three induction techniques; (3) employment of a favorite place imagery and progressive relaxation while in hypnosis in order to achieve relaxation; and (4) development of imagery intended to resolve the insomnia (Table 2). When appropriate, imagery regarding other symptoms was suggested. Patients were encouraged to practice self-hypnosis at bedtime on a nightly basis for at least 2 weeks, and on as needed basis thereafter.

**Table 2:** Examples of imagery intended to resolve insomnia

Imagine spending time in your relaxation place until you become tired.

Imagine going to sleep, and notice how you become tired and can fall asleep more easily as you watch yourself falling asleep.

Imagine a master control room in which there are switches or levers that control everything that you do. Find the control panel for your sleep. Change the control panel slowly towards the sleep position, and notice how you become sleepy as this occurs.

While you are in hypnosis, tell yourself that you are becoming sleepy.

If the insomnia did not resolve after the first session, patients were offered a second session during which they were taught how to use hypnosis in order to gain insight into potential stressors. Insight generating techniques included dream analysis, if the patients' sleep was disturbed by nightmares [10], talking about their stressors while in hypnosis, or hypnotic typing using the technique of automatic word processing [25]. Sleep hygiene education was provided to patients only if they reported during this session that their sleep was disturbed by environmental factors.

Provision of additional sessions was offered to patients with issues amenable to clinical hypnosis other than insomnia.

Formal testing for psychological disorders was not utilized as such testing is not used routinely at our Pulmonary Center. Hypnotizability was not assessed because there is little evidence to support the clinical utility of hypnotizability scales with children [20].

This report involved a retrospective chart review without identification of patients, thus exemption was given from review by the SUNY Upstate Medical University Institutional Review Board.

## Results

The 75 children's stated reasons for insomnia are listed in Table 3. Younger children were more likely to report that the insomnia was related to their fears (t-test, p < 0.001). Following the development of insight using hypnosis, some children concluded that their worries might be addressed with the thoughts and plans listed in Table 4.

**Table 3:** Children's stated reasons for their insomnia n = 75

	Number reporting	Average age (yrs.) +/- S.D.
Stressed by academic issues	21	13.1 +/- 2.1
Fears (being alone, darkness, dying, nightmares, or kidnapping)	19	10.4 +/- 2.4
Stressed by expectations of parents	15	12.6 +/- 2.0
Stressed by parental separation or divorce	11	12.5 +/- 2.7
Difficulty with peers	9	13.4 +/- 3.4
Environmental distraction (e.g., noise in bedroom)	4	10.3 +/- 1.9
Abuse in early childhood	4	13.3 +/- 1.9
Worries about own health	3	12.0 +/- 1.0
Recent move of child with family	3	11.7 +/- 2.5
Loss of significant person (as a result of move or death)	3	11.7 +/- 3.8
Can't stop thinking	2	14.0 +/- 4.2
Upset they cannot fall asleep	2	12.0 +/- 1.0
Blames self for maternal suicide	1	12
Worried about sexual orientation	1	12
		
Reasons cited only following use of hypnosis for insight		
		
Worried about safety of family	5	13.2 +/- 1.6
Worried about growing up	2	14.0 +/- 1.6
Too sensitive to other people's fears	1	12

**Table 4:** Children's thoughts and plans following development of insight regarding their stressors n = 19

	Number reporting
Their fears about their family's safety were unfounded	4
Tutoring at school for a specific subject would be helpful	4
Discussion regarding parents' expectations with their parents would be helpful	3
They should talk with their friends about their feelings	3
They should "go with the flow" rather than become distressed by events	2
Their friends can be trusted	1
They have an ability to control what worries them	1
They should be "more positive"	1

One session of hypnosis instruction was provided to 35% of the patients, two sessions to 33%, and three or more sessions to 32%. All of the patients demonstrated successful use of hypnosis induction techniques and favorite place imagery during their first hypnosis instruction session. Follow-up after hypnosis instruction was provided for less than a month in 23% of the cases, for 1–3 months in 33%, and for more than 3 months in 44%.

Of the 70 patients reporting difficulty with sleep onset, 77% reported nightly difficulty, 17% reported difficulty 4–6 nights/week, and 6% 2–3 nights/week. Table 5 shows the baseline and outcome data of these patients within a month of the last hypnosis session based on whether the referral diagnosis was insomnia without an associated diagnosis, insomnia with anxiety or attention deficit disorder only, or insomnia associated with a medical condition. Only 10% of the patients reported no reduction in the time required for sleep onset.

**Table 5:** Efficacy of hypnosis for patients with sleep onset delay n = 70

	Reported time required for sleep onset			
	< 30 min.	30–59 min.	60–119 min.	> = 120 min.

Insomnia without associated diagnosis; n = 11				
Baseline	0%	18%	27%	55%
After hypnosis	82%	9%	0%	9%
Associated with anxiety or ADD* only; n = 12				
Baseline	0%	25%	33%	42%
After hypnosis	67%	25%	8%	0%
Associated with other diagnoses; n = 47				
Baseline	0%	26%	45%	28%
After hypnosis	74%	15%	9%	2%

* Attention deficit disorder

Of the 21 patients reporting nighttime awakenings, including 16 who also had difficulty with sleep onset, 23% reported nightly awakenings, 33% reported awakenings 4–6 nights/week, and 43% 2–3 nights/week. Among these patients, 52% reported resolution and an additional 38% reported improvement of the awakenings within a month of the last hypnosis session. No patients reported recurrence or worsening of insomnia after the first month following the hypnosis intervention among the 77% who were followed for more than a month

Somatic complaints amenable to hypnosis were reported by 41% of the patients. These symptoms included chest pain, dyspnea, functional abdominal pain, habit cough, headaches, and vocal cord dysfunction. Among these patients, 87% reported improvement or resolution of the somatic complaints following hypnosis.

## Discussion

In this report, insomnia resolved in the majority of the patients after one or two instruction sessions. Thus, in order to achieve a high rate of improvement, it appears that fewer therapy sessions involving hypnosis may be required than has been suggested for adults [16-18]. The improvement rate in this study is similar to the 85% success rate reported in controlled studies of combinations of behavioral interventions for sleep disturbances in children [15].

Self-hypnosis may be of benefit for insomnia because of its effects in reducing physiologic arousal [20]. Additionally, the patients may have improved because their pre-sleep thoughts were altered by the employed hypnotic imagery and/or derived insights. The new thought patterns may have redirected patients from their usual anxiety provoking thoughts, thus leading to more calm [26]. Further, sleep disturbances may have been reduced as a result the high rate of improvement in the patients' somatic complaints following use of hypnosis.

The children's frequent report that their insomnia resulted from issues relating to school, parents, peers, and fears is similar to previous reports of common thought patterns in children with insomnia [27], as well as in adults who report intrusive and worrisome thoughts as the most common reason for their insomnia [28]. Use of hypnosis as a way to generate insight may have been helpful in resolving the insomnia for some of the children.

Patients with medical diseases may be more prone to insomnia because they spend more awake time in their bedrooms as result of their illnesses. Thus, they learn to associate the bedroom with non-sleep activities [29]. Also, these individuals may worry about their health and impact of poor sleep on their condition. These thoughts may further aggravate their insomnia [29]. It is notable that in this study those who reported insomnia in association with other medical conditions improved at the same rate as those with insomnia only.

It is unlikely that the results of this study were attributable to chance because the average duration of insomnia was 3 years, while the reported improvement occurred within a month of the intervention. However, in the absence of a controlled study, it is not possible to conclude that improvement of the patients' insomnia required an hypnosis intervention or generation of insight. Potential confounders include the attention patients received as part of the therapeutic interaction, or effects of relaxation or derived insight independent of the hypnotic state. Further, the patients' self-reports regarding their insomnia and changes in their symptoms may have been inaccurate. For example, patients with insomnia often underestimate the time they sleep, overestimate the amount of time it takes them to fall asleep, and can misperceive sleep as wakefulness [29]. Also, patients may have reported improvement in order to please their physician. Thus, future studies of the effects of hypnosis in children would benefit from use of control groups, as well as systematic objective assessment of the patients' sleep patterns, such as based on parental observation, or at least utilization of patient sleep diaries that are kept contemporaneously during the time of study [28]. Additionally, future studies might include patients reporting poor quality sleep, without associated sleep onset delay or nighttime awakening, who were excluded from the current study, as well as systematic follow-up periods of longer than a month following hypnosis.

Hypnosis techniques can be used for pre-school children. However, they need to be modified appropriately for the child's developmental age [30]. Further, hypnosis for insomnia with pre-school children should take into account that causes of insomnia in young children usually are different than for older children [27]. For example, sleep problems of pre-school children are more likely to involve issues related to interactions with their parents [27].

Prior to initiation of cognitive behavioral therapy for insomnia it should be determined that the sleep problem is not a symptom of another medical problem such as obstructive sleep apnea or a seizure disorder [15]. The choice of therapy most appropriate for an individual patient should be based on the reason for insomnia as well as the child's ability to cooperate with the therapy [9].

## Conclusion

This report demonstrates that instruction in hypnosis, and insight derived from its use, appear to facilitate efficient therapy for insomnia in school-age children as young as 7 years.

## Competing interests

The author(s) declare that they have no competing interests.

## Authors' contributions

RA is the pediatric pulmonologist described in this report. He conceived the study and wrote the manuscript. MS collected and analyzed the data. Both authors read and approved the final manuscript.

## Pre-publication history

The pre-publication history for this paper can be accessed here:



## References

[B1] Silber MH (2005). Chronic insomnia. N Engl J Med.

[B2] Glaze DG (2004). Childhood insomnia: why chris can't sleep. Ped Clin N Amer.

[B3] Stores G (1996). Practitioner review: assessment and treatment of sleepdisorders in children and adolescents. J Child Psychol Psychiatry.

[B4] Johnson EO, Chilcoat HD, Breslau N (2000). Trouble sleeping and anxiety/depression in childhood. Psychiatry Res.

[B5] Miser AW, McCalla J, Dothage JA, Wesley M, Miser JS (1987). Pain as a presenting symptom in children and young adults with newly diagnosed malignancy. Pain.

[B6] Mindell JA, Owens JA, Carskadon MA (1999). Developmental features of sleep. Child Adolesc Psychiatr Clin N Am.

[B7] Blader JC, Koplewicz HS, Abikoff H, Foley C (1997). Sleep problems of elementary school children. A community survey. Arch Pediatr Adolesc Med.

[B8] Knutson K (2005). The association between pubertal status and sleep duration and quality among a nationally representative sample of US adolescents. Am J Hum Biol.

[B9] Navelet Y (1996). Insomnia in the child and adolescent. Sleep.

[B10] Linden JH, Bhardwaj A, Anbar RD (2006). Hypnotically enhanced dreamingto achieve symptom reduction: a case study of 11 children and adolescents. Am J Clin Hypnosis.

[B11] Mercer PW, Merritt SL, Cowell JM (1998). Differences in reported sleep need among adolescents. J Adolesc Health.

[B12] Carskadon MA, Acebo C (2002). Regulation of sleepiness in adolescents: update, insights, and speculation. Sleep.

[B13] Glaze DG, Rosen CL, Owens JA (2002). Toward a practical definition of pediatric insomnia. Curr Ther Res Clin Exp.

[B14] Reed MD, Findling RL (2002). Overview of current management of sleep disturbances in children. I: Pharmacotherapy. Curr Ther Res Clin Exp.

[B15] Owens JA, Palermo TM, Rosen CL (2002). Overview of current management of sleep disturbances in children. II: Behavioral interventions. Curr Ther Res Clin Exp.

[B16] Anderson JAD, Dalton ER, Basker MA (1979). Insomnia and hypnotherapy. J Royal Soc Med.

[B17] Stanton HE (1989). Hypnotic relaxation and the reduction of sleep onset insomnia. Int J Psychosomatics.

[B18] Becker PM (1993). Chronic insomnia: outcome of hypnotherapeutic intervention in six cases. Am J Clin Hypnosis.

[B19] Jacobs L (1964). Sleep problems of children: treatment by hypnosis. NY State J Med.

[B20] Olness K, Kohen DP (1996). Hypnosis and Hypnotherapy with Children.

[B21] Anbar RD (2001). Self-hypnosis for management of chronic dyspnea in pediatric patients. Pediatrics.

[B22] Anbar RD (2001). Self-hypnosis for treatment of functional abdominal pain in childhood. Clin Pediatr.

[B23] Anbar RD (2002). Hypnosis in pediatrics: applications at a pediatric pulmonary center. BMC Pediatr.

[B24] Anbar RD, Geisler SC (2005). Identification of children who may benefit from self-hypnosis at a pediatric pulmonary center. BMC Pediatr.

[B25] Anbar RD (2001). Automatic word processing: a new forum for hypnotic expression. Am J Clin Hypnosis.

[B26] Harvey AG, Payne S (2002). The management of unwanted pre-sleep thoughts in insomnia: distraction with imagery versus general distraction. Behav Res Therapy.

[B27] Howard BJ, Wong J (2001). Sleep disorders. Pediatr Rev.

[B28] Harvey AG, Tang NKY, Browning L (2005). Cognitive approaches to insomnia. Clin Psychol Rev.

[B29] Smith MT, Huang MI, Manber R (2005). Cognitive behavioral therapy for chronic insomnia occurring within the context of medical and psychiatric disorders. Clin Psych Rev.

[B30] Sugarman LI (1996). Hypnosis: teaching children self-regulation. Pediatr Rev.

